# “Rest of the folks are tired and weary”: The impact of historical lynchings on biological and cognitive health for older adults racialized as Black

**DOI:** 10.1002/alz70860_107731

**Published:** 2025-12-23

**Authors:** Paris AJ Adkins‐Jackson, Cesar Higgins Tejera, Dejania Cotton‐Samuel, Carla L Foster, Lauren L Brown, Kenjus T Watson, Tiffany N Ford, Tahlia Bragg, Betselot Wondimu

**Affiliations:** ^1^ Columbia University Mailman School of Public Health, New York, NY, USA; ^2^ Johns Hopkins University, Baltimore, MD, USA; ^3^ G.H. Sergievsky Center, Vagelos College of Physicians and Surgeons, Columbia University, New York, NY, USA; ^4^ Department of Neurology, Vagelos College of Physicians and Surgeons, Columbia University, New York Presbyterian Hospital, New York, NY, USA; ^5^ Columbia University, New York, NY, USA; ^6^ Leonard Davis School of Gerontology, University of Southern California, Los Angeles, CA, USA; ^7^ American University, Washington, DC, USA; ^8^ University of Illinois Chicago, Chicago, IL, USA; ^9^ Boston University CTE Center, Boston, MA, USA

## Abstract

**Background:**

Childhood structural racism may lead to poorer health and longevity for individuals racialized as Black. Racism‐related stress cumulatively taxes the body resulting in worsening biological and cognitive health. This study examines the association between state‐level exposure to historical lynchings (adverse childhood racism for modern older adults), with C‐reactive protein (CRP, a marker of systemic inflammation), and global cognitive performance (modified TICS).

**Method:**

We linked the percentage of lynchings of people racialized as Black at the state‐level between 1882 and 1968 from the Archives at Tuskegee Institute with repeated CRP and cognitive test scores at baseline (2006/2008), year 4 (2010/2012), and year 8 (2014/2016) for a national sample of older adults in the Health and Retirement Study (*N* = 10,500, aged >50). In multivariable generalized estimating equation models, we compared participants (by racialized group) living in states with high lynching proportions (>50th percentile) on changes in CRP and cognitive test scores adjusting for demographics, health conditions, and behaviors.

**Result:**

Mean age was 69 (SD = 9.9) and most participants were cisgender women (59%). On average participants racialized as non‐LatinX Black living in states with high lynching proportions experienced 18.5% (95% CI 3%, 36%) higher CRP levels and ‐0.92 (95% CI ‐1.34, ‐0.50) lower cognitive test scores than participants racialized as non‐LatinX Black that lived in states with lower lynching proportions.

**Conclusion:**

As artist Marvin Gaye sang in Flyin' High (in the Friendly Sky), “Rest of the folks are tired and weary,” which describes how adverse childhood racism is associated with inflammation and dementia risk for people racialized as Black.

